# Imaging of Musculoskeletal Soft-Tissue Infections in Clinical Practice: A Comprehensive Updated Review

**DOI:** 10.3390/microorganisms10122329

**Published:** 2022-11-25

**Authors:** Paolo Spinnato, Dakshesh B. Patel, Maddalena Di Carlo, Alessandra Bartoloni, Luca Cevolani, George R. Matcuk, Amandine Crombé

**Affiliations:** 1 Diagnostic and Interventional Radiology, IRCCS Istituto Ortopedico Rizzoli, via GC Pupilli 1, 40136 Bologna, Italy; 2 Department of Radiology, Keck School of Medicine, University of Southern California, Los Angeles, CA 90007, USA; 3 Imaging Department, Bambino Gesù Children’s Hospital, IRCCS, 00146 Rome, Italy; 4 Department of Orthopaedic Oncology, IRCCS Istituto Ortopedico Rizzoli, 40136 Bologna, Italy; 5 Department of Imaging, Cedars-Sinai Medical Center, Los Angeles, CA 90048, USA; 6 Department of Musculoskeletal Imaging, Pellegrin University Hospital, 33076 Bordeaux, France

**Keywords:** magnetic resonance imaging, ultrasonography, interventional, radiography, multidetector computed tomography, soft tissue infections

## Abstract

Musculoskeletal soft-tissue infections include a wide range of clinical conditions that are commonly encountered in both emergency departments and non-emergency clinical settings. Since clinical signs, symptoms, and even laboratory tests can be unremarkable or non-specific, imaging plays a key role in many cases. MRI is considered the most comprehensive and sensitive imaging tool available for the assessment of musculoskeletal infections. Ultrasound is a fundamental tool, especially for the evaluation of superficially located diseases and for US-guided interventional procedures, such as biopsy, needle-aspiration, and drainage. Conventional radiographs can be very helpful, especially for the detection of foreign bodies and in cases of infections with delayed diagnosis displaying bone involvement. This review article aims to provide a comprehensive overview of the radiological tools available and the imaging features of the most common musculoskeletal soft-tissue infections, including cellulitis, necrotizing and non-necrotizing fasciitis, foreign bodies, abscess, pyomyositis, infectious tenosynovitis, and bursitis.

## 1. Introduction

Musculoskeletal soft-tissue infections include a wide range of clinical conditions that are commonly encountered in both emergency departments and non-emergency clinical settings. These infections may occur in all age groups with a predilection for the elderly [1,2,3]. There are several well-known predisposing conditions, such as extremes of age, immunosuppression/systemic diseases, illicit drug abuse, alcoholism, peripheral vascular disease, trauma, burns, surgery, obesity, and malnutrition [1,4,5,6]. Moreover, some soft-tissue infections are limited to specific geographical areas with warm and humid climates.

A wide range of microorganisms responsible for these infections are usually found in polymicrobial combinations. Both aerobic and anaerobic bacteria can be present at the same time. In several cases, soft-tissue infections can be caused by a single microorganism (e.g., hemolytic Streptococcus spp, Staphylococcus spp, and Clostridium perfringens). The most common polymicrobial infections are caused by combinations of Staphylococcus, Streptococcus, Enterococcus, Enterobacteriaceae, and Bacteroides species [1,7]. Fungi and other atypical organisms can cause these infections too. The most frequent of these latter organisms is candida spp [7].

The most frequent soft-tissue infection subtypes include infectious cellulitis, fasciitis, infective tenosynovitis/bursitis, pyomyositis, and infected myonecrosis [1].

The most common clinical signs of soft-tissue infections are fever, malaise, local pain, oedema, erythema, palpable mass, and sometimes crepitus due to the presence of soft tissue gas [8]. Anyway, these clinical signs are usually scarce or even not present. 

Since clinical signs, symptoms, and even laboratory tests (e.g., leukocytosis, C-reactive protein, and erythrocyte sedimentation rate) of musculoskeletal soft-tissue infections can be unremarkable or non-specific, imaging plays a key role in many cases [1,2].

The aim of this review article is to provide a practical overview of the imaging features of the main musculoskeletal soft-tissue infections.

## 2. Imaging of Soft-Tissue infections: General Considerations 

**Figure 1:**
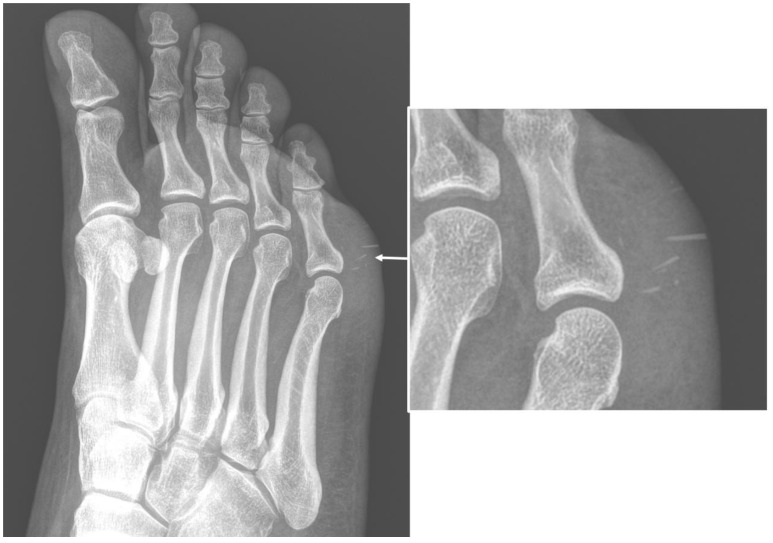
Conventional radiography (anterior–posterior oblique projection) of the right foot in a 44-year-old woman, who were presented to the emergency department with complaints about worsening pain and swelling in the plantar external region. The examinations confirmed local swelling of soft tissue and revealed the presence of thin foreign bodies, sea urchin quills (arrows and in enlargement), which was confirmed after surgical excision and anamnestic confirmation.

Imaging is a key element in characterizing soft-tissue infections, with the aim of confirming clinical suspicion and identifying the location and extent of involvement. Although it is rarely diagnostic, conventional radiography (CR) is often the initial modality in suspected soft-tissue infections [2]. CR can detect or confirm a local thickening of soft-tissue, and identify or exclude the presence of foreign bodies (Figure 1). 

**Figure 2:**
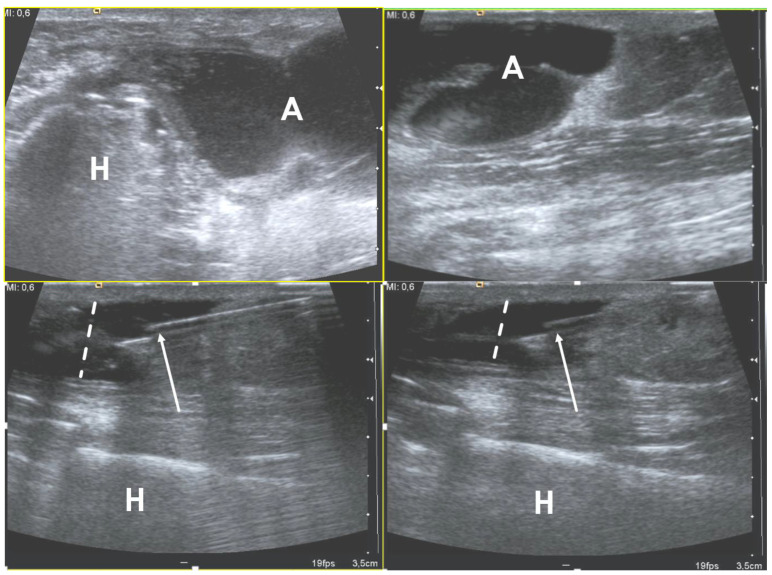
Ultrasound examination of a soft-tissue abscess of lateral aspect of the left harm in a patient who were recently operated for humeral fracture. The needle’s tip is placed inside the abscess (arrows) under ultrasound guidance (in-plane approach); during the fluid aspiration, the reduction of the abscess can be appreciated (dotted lines). H = humerus, A = abscess.

Magnetic resonance imaging (MRI) is the most sensitive imaging tool for the assessment of soft-tissue infections due to its resolution, optimal inherent contrast, and great anatomical details that permit the accurate evaluation of the extent of tissue involvement [2]. Computed tomography (CT) may be useful for the assessment of soft-tissue infections in several conditions including infections with gas components (e.g., necrotizing infections) and for the detection of foreign bodies. Ultrasound (US) can be particularly useful for non-invasive diagnosis of soft-tissue infections and collections (e.g., abscesses and joint effusions). Moreover, US is widely used as a guidance for percutaneous needle aspiration of soft-tissue collection (Figure 2), offering the possibility to perform microbiological analyses on the specimens [2].

The following sections will provide in-depth information regarding the imaging features of the most common soft-tissue infections.

### 2.1. Cellulitis 

Cellulitis is an acute infection of the skin and the subcutaneous fat and may present without or with systemic signs of infection. It can involve the superficial fascia but does not involve the deep fascia. Infection follows a visible or invisible break in the skin surface, with Streptococci and Staphylococcus aureus being the most common causative organisms [9,10]. The risk factors include cutaneous lesions and wounds, diabetes, immunocompromised states, vascular insufficiency, and foreign bodies [9,11]. Skin erythema, swelling, warmth, tenderness, and lymphadenopathy are common in clinical presentation [10]. It is a clinical diagnosis, but imaging may be performed when there is rapid progression, systemic symptoms, or concern for deeper involvement [9]. The aim is to exclude deeper extension and abscess formation [12]. Imaging can also show foreign bodies (see later). Uncomplicated cellulitis is managed medically with antibiotics.

Imaging is usually not performed, but plain radiographs are often obtained. The findings are nonspecific, showing diffuse soft tissue swelling and increased density of the subcutaneous fat with a loss of fat planes [13]. It can detect soft tissue gas which is seen in necrotizing infections. 

Ultrasound (US) has commonly been used as a front-line examination and shows subcutaneous soft tissue edema, increased echogenicity of the subcutaneous tissues, hypoechoic fluid insinuating, and separation of fat lobules that gives a cobblestone pattern [2,13]. With color or power doppler, increased vascularity may be seen. US can identify abscess, which is seen as an anechoic or hypoechoic collection with echogenic rim and increased peripheral vascularity, but not internal vascularity [13]. US is also often used to exclude deep venous thrombosis which can clinically mimic cellulitis. 

Computed tomography (CT) with intravenous iodinated contrast has also been used to evaluate cellulitis and shows skin thickening, underlying fat stranding or subcutaneous edema, and thickening of underlying fascia [13,14]. Enhancement of the septations and asymmetric distribution differentiate it from bland edema as can be seen with cardiac or renal failure. Abscess, if present, is seen as a ring-enhancing fluid collection. 

Magnetic resonance imaging (MRI) shows a similar appearance with skin thickening and increased signal in subcutaneous fat on fluid-sensitive sequences [2,15]. The increased signal may be streaky (fat stranding) or diffuse, and there is a corresponding low signal on T1 weighted sequence [1,12]. Edema-like signal can be seen extending along the fascia [2]. Following the administration of intravenous gadolinium-based contrast material, there is diffuse enhancement [13] that is similar to CT and helps differentiate cellulitis from noninfectious causes of edema, such as cardiac or renal insufficiency [11,14]. An abscess, when present, will be seen as intermediate to low T1 and high fluid-sensitive signal collection with peripheral enhancement after the intravenous contrast administration [16].

### 2.2. Necrotizing and Non-Necrotizing Fascitiis 

Infective fasciitis is an infection of the fascia and can be non-necrotizing (NNF) or necrotizing. Necrotizing fasciitis (NF), as a part of necrotizing soft-tissue infections (NSTI), is a rare but rapidly progressive infection of the deep soft tissue and fascia. If not treated promptly, it can lead to high morbidity and mortality [10,17]. Early surgical intervention is an important prognostic factor [10].

This infection is commonly presented as type 1, which is polymicrobial with common organisms that include aerobes and anaerobes. It commonly affects the perineum and trunk and is common in patients of advanced ages who have decreased immunity from preexisting diseases. Type 2 disease is monomicobial with Group A Streptococcus (most common) or staphylococcus aureus (including methicillin-resistant Staphylococcus aureus) being the common organisms. Patients are usually younger with trauma being a common method of inoculation. Vibrio and Clostridium species (type 3) and fungi (type 4) are additional causative organisms [10,18]. Predisposing factors include diabetes, obesity, immunosuppressed states, trauma (which can be minor, such as insect bites), intravenous drug use, venous insufficiency, and peripheral vascular disease.

Common sites of infection include the extremities, perineum, trunk, and head and neck. NF has been given specific names when affecting certain regions—Ludwig angina when it affects the submandibular region and Fournier gangrene when it affects the perineum [19]. 

This infection commonly follows a penetrating injury or surgery, but can also occur without a history of trauma [17,19]. Following inoculation, there is a rapid growth of bacteria with production of exotoxins and activation of cytokines. Blood vessels are thrombosed and damaged with resultant ischemia and necrosis. The infection spreads along the fascia, far and wide, and can affect all tissues from skin to muscles [2,10,19]. Necrotizing soft-tissue infection (NSTI) is an advocated terminology to encompass all necrotizing infections deep to the skin, including the fascia [2,11,20].

The diagnosis of NF is clinical and requires a high index of suspicion. At surgery, there are dull friable fascia with lack of resistance, necrotic tissue without pus, and dishwater exudate [17,19,21]. There is a wide variation in clinical presentation, but patients often present with signs and symptoms similar to cellulitis. This includes soft-tissue swelling, erythema, pain, tenderness, and fever [18]. Pain that is out of proportion to clinical findings, tenderness extending beyond the region of skin manifestation, and wooden hard feel of the subcutaneous tissues are important clinical indicators of deep involvement. As the disease worsens, the skin becomes discolored with formation of bullae (often hemorrhagic) that slough as the gangrene progresses. These are accompanied by signs of systemic infection that progress to shock with hypotension and multiorgan failure [19]. Crepitus may be felt as if there is soft tissue gas which forms with polymicrobial and clostridial infections [10]. Toxic shock syndrome may be seen with Streptococcal infection, further adding to the disease severity [18].

The initial presentation may be confusing, and the major challenge is differentiating superficial from deep infection and distinguishing non-necrotizing infection from necrotizing fasciitis. A high degree of clinical suspicion is needed as there can be rapid deterioration in NF. Laboratory tests have been used as an adjunct to diagnosis. Laboratory Risk Indicator for Necrotizing fasciitis (LRINEC) score is a useful tool comprising scores from common laboratory tests, including C-reactive protein, white blood cell count, hemoglobin, sodium, glucose, and creatinine [22]. Based on the total score, the suspicion for NF is stratified into low, intermediate, or high risk. A score of six or higher is highly suspicious for NF. Although initial reports have indicated high sensitivity, later studies have shown lower sensitivity [23] and a lower score does not exclude NF [18]. The sensitivity and specificity of LRINEC score of six were only 68.2% and 84.8%, respectively, in a recent systematic review and meta-analysis [23].

Imaging can aid management but should not preclude early intervention [10]. It delineates the extent of the disease to guide debridement [24]. 

Plain radiographs are easy to obtain but are commonly nonspecific and show soft tissue swelling. The presence of gas is specific but is seen in less than half or even less than one third of the patients [17,25]. Its absence does not exclude NF as it may not be present early in the disease, and many organisms do not produce gas. In a recent systematic review and meta-analysis, the specificity of plain radiography was 94% but the sensitivity was only 49% [23]. Differential includes gas gangrene and gas tracking along the fascial planes from the ulcer. 

There have been several articles promoting the usefulness of US for the diagnosis of NF [26]. US, although easily available, is limited by operator dependence and experience, and there are limited data about its utility [26,27,28,29]. It shows diffuse thickening and increased echogenicity of the subcutaneous soft tissue, gas, and perifascial fluid (mnemonic STAFF—Subcutaneous thickening, air, and fascial fluid). Although initially encouraging, a more recent study, using a cut-off thickness of 2 mm for fascial fluid, had relatively low sensitivity and specificity of 75% and 70%, respectively [30]. Gas is seen as echogenic focus with dirty posterior acoustic shadowing. Gas precludes the evaluation of deeper structure and may underestimate the extent of disease. Point-of-care US can be fast but may not be available universally [31]. In a recent single-institute study, point-of-care US was reported to have very high sensitivity, specificity, and negative predictive value for the diagnosis of NF of 100%, 98.2%, and 100%, respectively, but this needs to be confirmed with additional studies [26]. 

CT scan is fast but involves ionizing radiation. Although CT with contrast has been recommended, this may not be possible in the setting of acute renal failure. When present, gas is more easily demonstrated than plain radiographs. In a recent review, the presence of gas in CT had a pooled sensitivity and specificity of 48.6% and 93.2%, respectively [32]. In addition to gas along the fascia, other common CT findings include increased density and stranding (edema) in the subcutaneous fat, edema and thickening of the superficial and deep fascia, blurring of the intermuscular fascial planes, nonenhancement of the fascia, and fluid collection [13,33]. Thickening and enhancement of the muscles may be seen [20]. There is a wide range of sensitivities and specificities for the diagnosis of NF with CT. In one study in an appropriate clinical setting, fluid along the deep fascia was the only CT parameter highly associated with necrotizing fasciitis, with sensitivity and specificity of 46.2% and 94.1%. It was found to be more useful than LRINEC score [34]. In NSTI, perifacial gas; muscle and fascial involvement; and fluid tracking in the subcutaneous tissues, lymphadenopathy, and subcutaneous edema are more common than in NNSTI [35]. Based on these findings, a CT scoring system has been proposed, with score of >6 favoring NSTI instead of NNSTI. Using this criterion, the sensitivity and specificity were found to be 86.3% and 91.5%, respectively [35]. These CT findings also need to be differentiated from causes of bland edema, such as renal failure and congestive heart failure. In NF, the findings are asymmetric, while, in bland edema, they are symmetric and diffuse.

MRI has superior soft tissue contrast resolution but may not be feasible when time is of essence. Fluid-sensitive sequences, such as T2 weighted fat suppressed (T2FS), short tau inversion recovery (STIR), and post-intravenous contrast T1-weighted fat suppressed (T1FS), are the most important sequences. Gas can be identified as signal void on all pulse sequences and shows blooming on gradient recalled-echo sequence. The other key finding in NF is a high fluid-sensitive signal along the deep peripheral fascia, which has pooled sensitivity and specificity of 86.4% and 65.2% [32]. The absence of a high fluid-sensitive signal along the deep fascia essentially excludes NF. Several other MRI features can be seen and include a thickening of the fascia, a deep extension of the high fluid-sensitive signal along the intermuscular fascia, an abnormal signal intensity in the muscle (patchy or band-like at the periphery), and nonenhancement in the region of high fluid-sensitive signal [36]. Increased fluid-sensitive signal, thickening of the fascia >3 mm, extensive involvement of the deep fascia, involvement of three or more compartments in one extremity, and focal or diffuse nonenhancement of fascia are more commonly seen in NF than in NNF [36]. There is mixed pattern at post contrast, with enhancement being secondary to extravasation of contrast due to increased capillary permeability [14], while nonenhancement is considered secondary to thrombosis of vessels and necrosis [1,14]. The fluid-sensitive sequences overestimate the extent of the disease by showing surrounding areas of reactive noninfectious edema [15]. On the other hand, post-contrast imaging may underestimate the disease by showing a lack of enhancement secondary to the occlusion of vessels [1,15,20,36]. 

To improve diagnostic accuracy, a new scoring system with contribution from MRI findings and LRINEC score has been proposed which shows improved sensitivity and specificity (96% and 60%, respectively), but it needs to be validated. The two MRI findings used are thickening of deep fascia >3 mm and multicompartment involvement, confirming these to be the two most important MRI findings [37]. In another study, an algorithmic approach was proposed to differentiate NF from cellulitis using T2 hyperintensity of intermuscular fascia and diffuse T2 hyperintensity of deep peripheral fascia [38]. This improves the sensitivity but, again, the results need to be validated.

Of note, the increased fluid-sensitive signal in the fascia is nonspecific and can be seen with NNF, cellulitis, prior radiation therapy, trauma, inflammatory myositis, and other conditions, such as eosinophilic fasciitis [25,39,40]. MRI abnormality that is limited to the subcutaneous tissues and superficial fascia or is limited to the peripheral portion of the deep fascia favors cellulitis over NF [39]. Diffuse T2 hyperintensity of the deep peripheral fascia and T2 hyperintensity of the intermuscular fascia, particularly if diffuse, favor NF over severe cellulitis [38]. In NNF, deep fascial involvement is usually thin (<3 mm), affecting fewer than three compartments, and, if the intermuscular fascia is involved, it is at the periphery [36]. Postoperative changes may closely mimic necrotizing fasciitis due to postoperative fluid tracking along the fascial planes, but these findings should resolve over time [20]. 

Diffuse hyperintense fluid-sensitive signal and enhancement, as well as intramuscular abscesses, are seen in pyomyositis, while peripheral band of hyperintensity and enhancement is seen in NF [39,41]. Fascial enhancement is seen in both but is thicker and uneven in pyomyositis [41].

In summary, prominent perifascial gas in soft tissue is virtually diagnostic of NF, but its absence does not exclude NF. Fluid along the deep peripheral fascia is seen in NF and is detected using US, CT, and MRI. Its absence excludes NF, but fluid along the deep fascia is also see in cellulitis and NNF. More severe and extensive involvement seen as thickening of the fascia of >3 mm, extensive involvement of the deep intermuscular fascia, and involvement of more than three compartments favor NF. The key findings on imaging are summarized in Table 1.

### 2.3. Soft-Tissue Foreign Body 

Each year, more than 11 million patients present to emergency departments with traumatic wounds and lacerations, with approximately 10% of these cases having a retained foreign body [42]. These retained foreign bodies are most commonly found in the extremities, with nearly one third involving the wrist, hand, or fingers [43]. The most common retained foreign bodies include wood, glass, and metal [44]. Soft tissue foreign bodies may also be the result of self-inflicted penetrating trauma, also known as self-embedding behavior [45]. Foreign bodies may also result from medical procedures, due to retained sponges or instruments or broken fragments of drains, implants, or surgical devices [46]. Metallic foreign bodies are common after gun-related injuries. Most foreign bodies are recognized at the time of injury, but more than one third may be missed at initial physical evaluation and can remain asymptomatic [47]. Although most foreign bodies can be removed by the injured patient without a problem, some cannot and are presented to the care of a physician. One series of foreign bodies in the hand reported a wide range of time anywhere from the day of injury to 20 years later for the removal of the retained foreign body (mean of seven months), with 43% being removed within one week and 11% being retained for more than one year [47].

Retained foreign bodies can result in a granulomatous immune response as the surrounding tissues attempt to wall off the foreign body from the patient, also known as foreign body reaction [48]. This starts with an aggregation of macrophages, lymphocytes, plasma cells, and fibroblasts that surround the foreign body. As most foreign bodies are too large to phagocytose, the macrophages undergo epithelioid transformation and fuse to form multinucleated giant cells and produce cytokines that stimulate fibroblasts to encapsulate the foreign body in a collagen matrix [49]. For organic foreign bodies, such as wood or animal parts, the foreign body reaction will continue until degradation; however, for nondegradable foreign bodies, the reaction continues until a well-formed capsule separates the foreign body from the immune system and may become asymptomatic for years or until trauma disrupts this capsule [50]. 

The first-line imaging modality for suspected foreign body should be conventional radiography, which is capable of detecting 80% of all foreign bodies [51,52]. This mostly depends on the atomic number and density of the foreign body; lower-density objects are radiolucent, but up to 98% of higher-density radiopaque objects are also depicted on conventional radiographs [44,52]. Metal, glass, stone, graphite, and calcified biologics (e.g., sea urchin spines) are all radiopaque, whereas wood, plastic, acrylics, and non-calcified biologics (e.g., larvae) are radiolucent [43,44].

**Figure 3:**
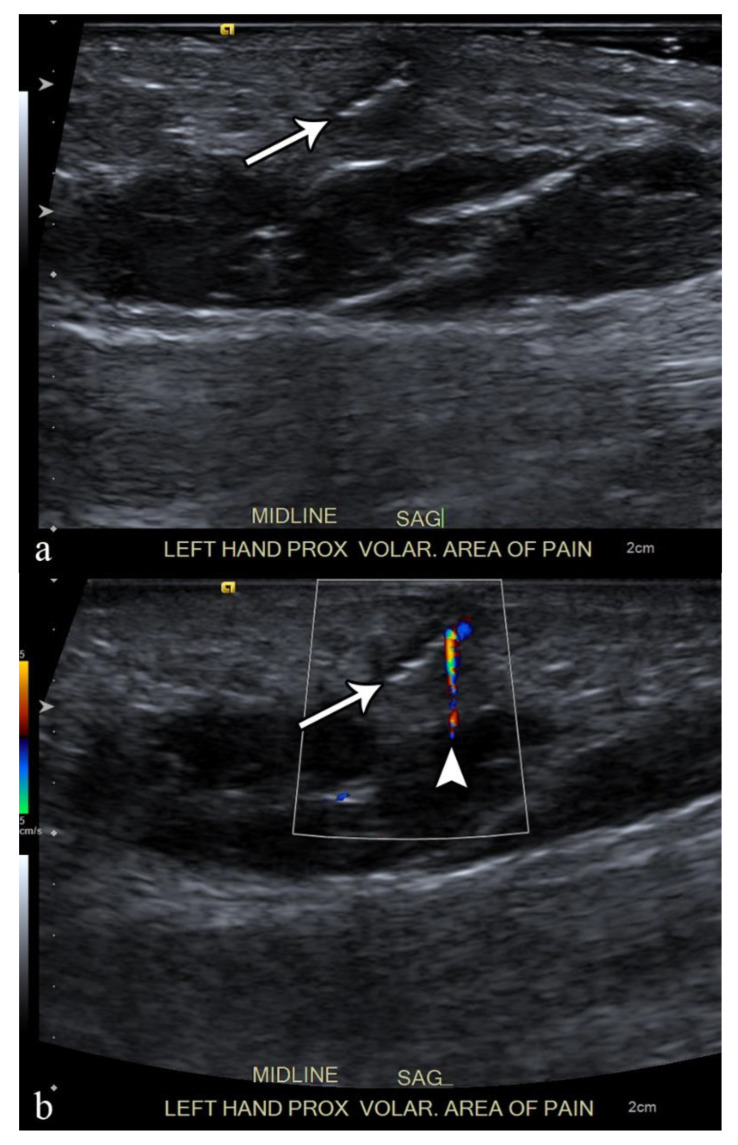
A 50-year-old man with left volar mid-palm pain after recent injury by glass. Grayscale US (**a**) demonstrates a 4 mm linear echogenic retained foreign body (arrows), with a surrounding hypoechoic halo (foreign body reaction/granuloma) in the subcutaneous tissues, and a color comet-tail artifact (arrowhead) on color Doppler (**b**).

Ultrasound is the best imaging modality for the evaluation of suspected radiolucent foreign bodies, with all foreign bodies demonstrating hyperechogenicity on ultrasound with posterior acoustic shadowing; foreign bodies with an irregular or curved surface demonstrate a “clean” shadow, and those with a flat or smooth surface, such as metal, glass, or plastic, demonstrate a “dirty” shadow with posterior reverberation (comet tail artifact) (Figure 3) [53,54,55]. 

Ultrasound also has the advantage of demonstrating surrounding foreign body reaction with a hypoechoic rim and hypervascularity on color or power Doppler imaging as early as 24 h after the penetration of the foreign body [56,57]. Ultrasound may also demonstrate complications related to the foreign body, including infection or vascular or tendinous injuries [44]. Although user-dependent, ultrasound also has the added advantage of being able to plan and provide real-time imaging-guidance for foreign body removal (Table 2), which, in a study by Tahmasebi et al., was possible in more than 50% of cases without short or long-term complications [46,58].

**Figure 4:**
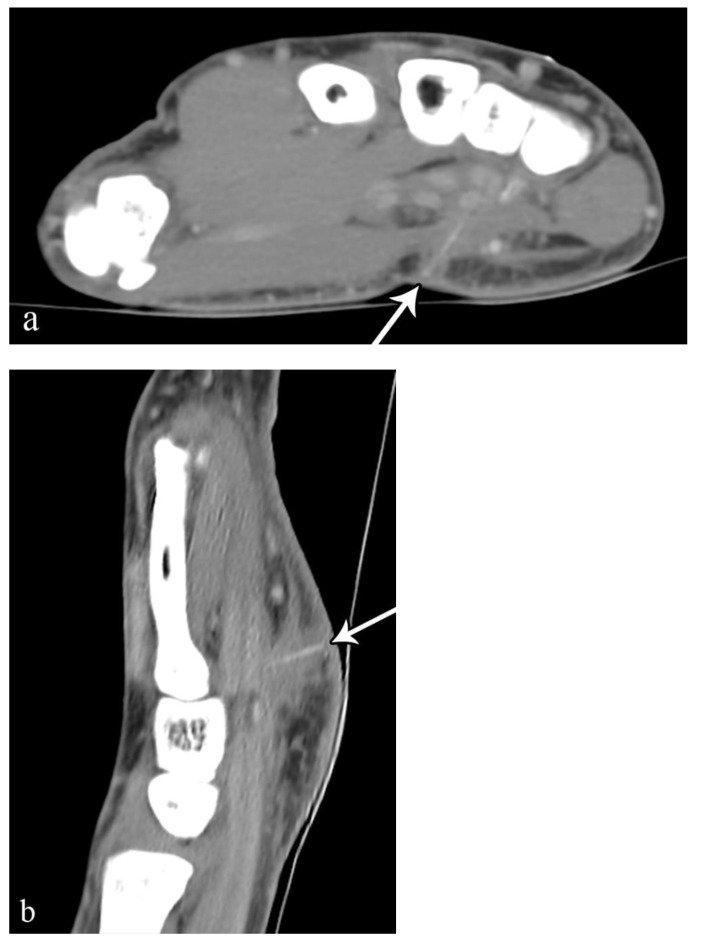
A 30-year-old man with right hand pain after climbing a telephone pole. Axial (**a**) and sagittal (**b**) CT images demonstrate a 1.4 cm linear density compatible with a wood splinter (arrows), extending from the palmar subcutaneous tissues to the flexor retinaculum.

Computed tomography (CT) can be useful for suspected foreign bodies not identified by radiography or US, particularly deep foreign bodies [43]. CT is 5–15 times more sensitive than radiography and can identify some radiolucent foreign bodies, including plastic and wood (Figure 4) [59,60].

**Figure 5:**
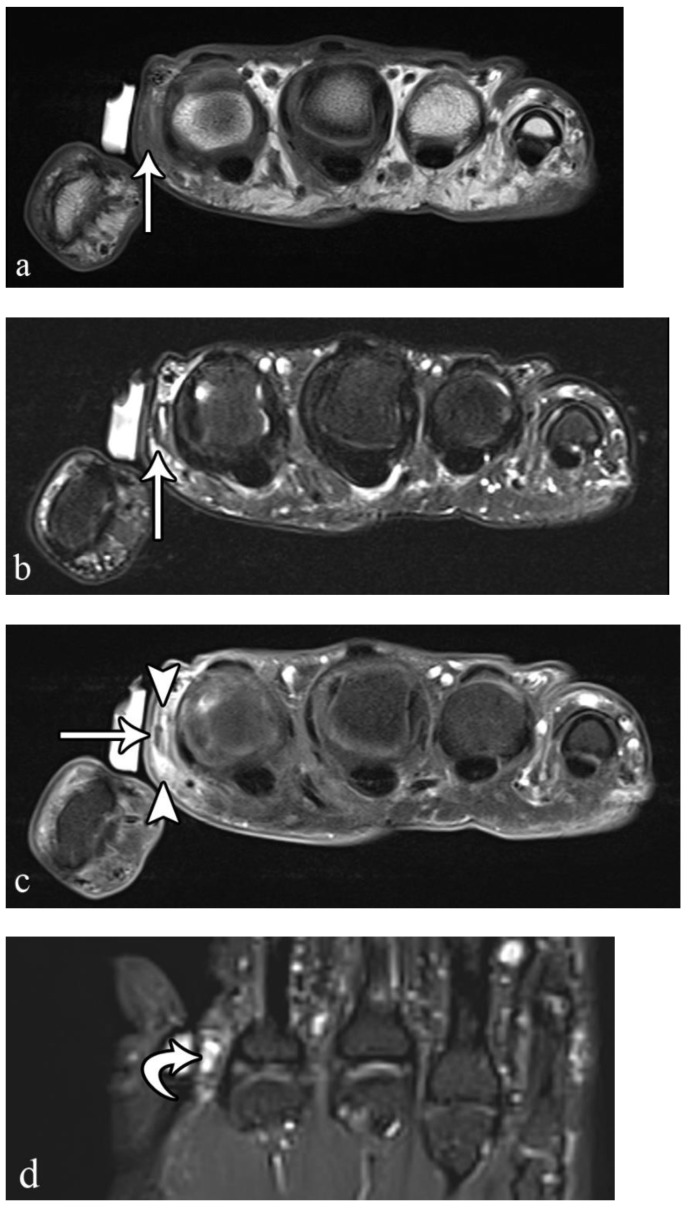
A 49-year-old man with left index finger pain and laceration after a wood-working injury at 6 weeks prior. Axial T1 **(a**), T2 FS (**b**), post-contrast T1 FS (**c**), and Cor PD FS (**d**) MRI demonstrate an 8 mm linear hypointense foreign body (arrows) with surrounding enhancement (arrowheads) and fluid signal intensity (curved arrow), compatible with a foreign body granuloma.

Dual-energy CT also shows promise by using material decomposition to allow for more specific differentiation of the type of foreign body [61]. Magnetic resonance imaging (MRI) is not as sensitive for foreign body detection, generally due to the thicker slices of the examination, but can appear as a discontinuous linear T1/T2 low-signal intensity structure with straight edges or right angles [43]. In the acute phase, there may be adjacent edema and peripheral enhancement, especially with biologic foreign bodies; in the chronic phase, granulomatous reaction can form a T1/T2 hypointense fibrous wall around the foreign body (Figure 5).

Metallic foreign bodies or associated soft tissue gas may have prominent susceptibility to artifacts, especially on gradient recalled echo (GRE) sequences [62]. The main advantage of MRI over other imaging modalities is the superior contrast resolution, which can be helpful for identifying complications of foreign bodies, including infection or injuries to tendons, ligaments, or neurovascular structures.

The appearance of various foreign bodies on different imaging modalities is summarized in Table 3 [43].

The most frequent complication of retained foreign bodies is infection, with an incidence of up to 14.8%, including cellulitis and soft tissue abscess; more serious deep infections, such as osteomyelitis and septic arthritis, are less common, occurring in up to 1.8% of patients [63]. The most common organism to cause infection is Staphylococcus aureus, with late presentation for treatment and biologic foreign bodies being associated with increased infection rates [47,63]. Unsuspected retained foreign bodies may be a cause of the failure of initial antibiotic therapy. MRI is the modality of choice when infection is suspected, demonstrating soft-tissue abscesses as thick-walled rim enhancing fluid collections with septations and debris and restricted diffusion on diffusion-weighted imaging (DWI) (3). MRI can also suggest septic arthritis, tenosynovitis, and bursitis with fluid distension, synovitis, and peripheral enhancement, although definitive diagnosis is made with image-guided aspiration. MRI also has a 95% specificity and a 91% sensitivity for diagnosing osteomyelitis, with a negative predictive value near 100%, and the classic findings of bone marrow T2 hyperintensity with corresponding T1 hypointensity [64].

### 2.4. Abscess

According to the Society of Skeletal Radiology (SSR), a soft-tissue abscess (STA) corresponds to a localized collection of pus resulting from the invasion of a pathogen, with a peripheral capsule created by macrophage, fibrin, and granulation tissue generally following an untreated phlegmon [65]. STAs can be acute or chronic, and deep-seated or superficial. When located intra-muscularly, STAs have also been named pyomyositis.

The origins of STAs can be direct inoculations due to skin-puncture injuries and foreign bodies and, less frequently, hematogenous spreading from a nearby infected site. They are predisposed by immunosuppression, diabetes, systemic diseases, malnutrition, drug abuse, obesity, extreme age, pre-existing cutaneous lesions, trauma, or surgery [1].

The most common pathogens correspond to Gram-positive cocci, especially Staphylococcus aureus (with methicillin resistance in nearly half of the cases) and hemolytic Streptococcus spp., but many germs are encountered, such as Clostridium spp, Enterococcus, Enterobacteriaceae, and Bacteroides spp or Pseudomonas aeruginosa [66,67]. However, most STAs are polymicrobial with a mixture of aerobic and anaerobic bacteria. 

Clinically, patients usually demonstrate fever, possibly sepsis, and painful, erythematous, and inflammatory swelling. Biologically, laboratory analysis shows increased leukocytes and C-reactive proteins. 

Conventional radiographs are of limited interest. They may show swelling, increased opacity, dystrophic calcification, and bull of gas and air–liquid level depending on the nature and chronicity of the STA. If the STA is close to the bone, a periosteal reaction may be seen [68]. 

On ultrasonography with Doppler, STAs display a central heterogeneous, hypoechoic, and non-vascularized collection and an irregular and highly vascularized pseudo-capsule, which is surrounded by edematous, hyperechoic, and hyperemic phlegmon tissues. Internal debris may be seen as small, swirling hyperechoic structures inside the collection and move depending on dynamic compression and the patient’s motion. Additionally, gas can be detected as a bright punctate echo with acoustic shadowing in the case of anaerobic bacteria [68,69,70,71]. Peripheral calcifications and marked liquefaction indicate a chronic STA. The location, dimension, septations, uniqueness or multiplicity, as well as the presence of gas and calcifications, should be detailed in the radiological report.

On CT, ideally performed with an intravenous contrast medium injection, STAs present as fluid attenuation, a collection circumscribed by an enhanced, irregular thin wall. Again, enhanced septa, gas, and calcifications can be seen but not systematically. The surrounding tissues can also demonstrate edema, cellulitis, and mild enhancement [72,73]. 

**Figure 6:**
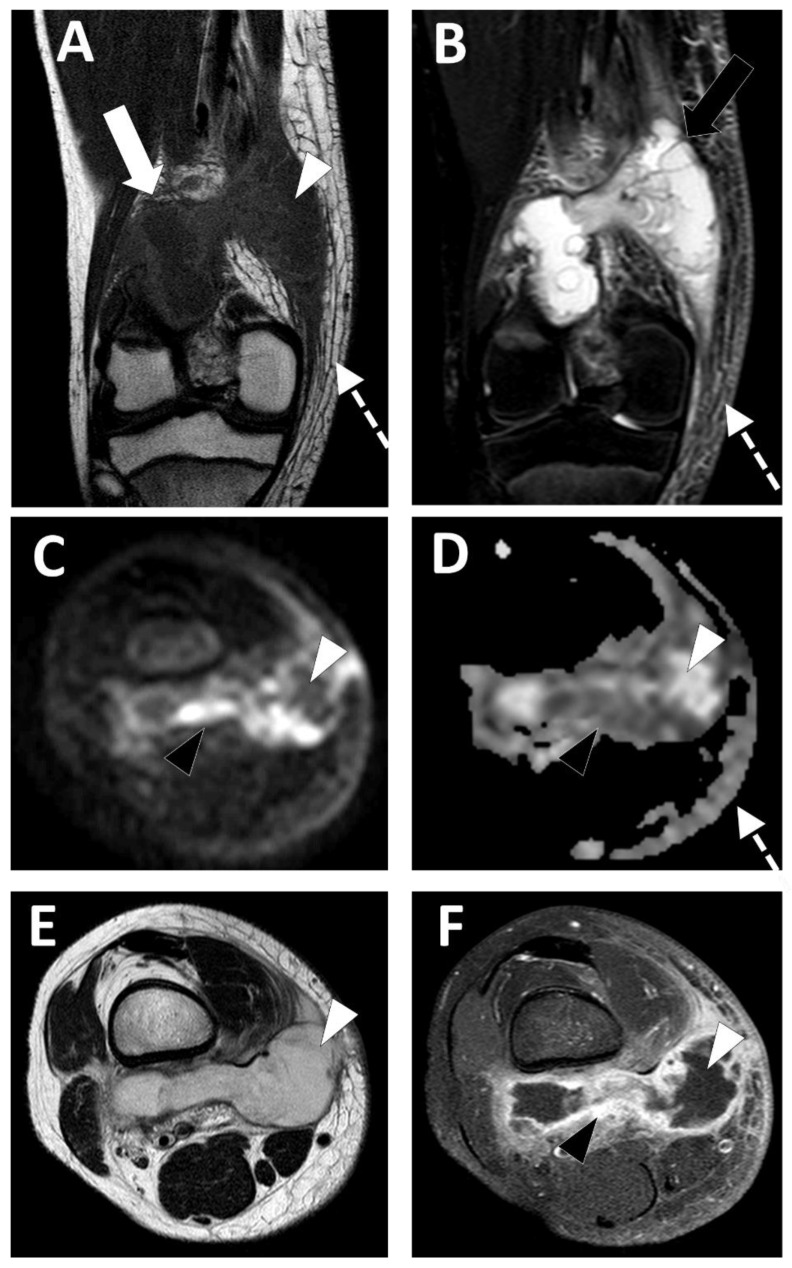
Deep-seated abscess of the right popliteal region in a 13.5-year-old boy presenting with a soft tissue painful swelling, fever, and inflammatory syndrome on blood analysis. The MRI comprises the following sequences: (**A**) coronal T1-weighted imaging (WI), (**B**) coronal T2-WI, (**C**) axial TRACE of diffusion MRI, (**D**) apparent diffusion coefficient (ADC) map, (**E**) axial T2, and (**F**) axial T1 with gadolinium chelate injection and fat suppression. On MRI, the abscess is presented as a fluid-like collection (white arrowhead) with high signal intensity (SI) on T2-WI, low SI on T1-WI, low SI on TRACE, and high ADC value and no contrast enhancement.

Conventional MRI with injection of gadolinium chelates provides the highest accuracy in the diagnosis and staging of STAs, with a sensitivity of 97% and a specificity of 77% (Figure 6) [74].

STAs demonstrate a centrum with low to intermediate signal intensity (SI) on T1-weighted imaging (WI) and fluid-like SI on T2-WI, and are surrounded by irregular but well-defined rim with contrast enhancement and edematous tissues. An additional MRI feature has been described in subacute to chronic STAs, namely the ‘penumbra sign’ which consists of the presence of a thin peripheral rim with high SI on pre-contrast T1-WI due to the active and highly vascular inflamed granulation in the surrounding tissue [75]. The penumbra sign can be useful to discriminate STAs from soft tissue neoplasms and, in such a setting, has demonstrated a specificity of 98% and a sensitivity of 54% [75]. Moreover, diffusion weighted imaging (DWI) could replace contrast agent injection according to Chun et al. to identify abscesses in patients with soft tissue infection, although an injection remains needed if there is any suspicion of neoplasm. The authors showed similar sensitivity and specificity in diagnosing STAs in a cohort of 119 patients with soft-tissue infection, i.e., 77.5–90% and 88.6%, respectively [76]. In more detail, in the case of acute to subacute STA, DWI can demonstrate water motion restriction inside the collection due to viscous pus and, thus, has a low apparent diffusion coefficient (ADC ≈ 0.6–0.11 × 10^−3^ mm^2^.s^−1^), which can be helpful to distinguish the STA from a soft tissue neoplasm [77]. More liquefied STAs display higher fluid-like ADC values (>2 × 10^−3^ mm^2^.s^−1^) circumscribed by a wall with restricted diffusion [77,78].

Some pathogens also display additional specific features. For instance, soft tissue involvement in alveolar echinococcosis (due to Echinococcus species, which are tapeworm parasites) is characterized by multiple cyst-like lobulated lesions with possible calcifications [79]. STAs in actinomycosis (due to Actinomyces species, an anaerobic Gram-positive bacterium) are rare, generally presenting in the abdominal wall or psoas with a digestive, urinary, or genital origin, and can demonstrate infiltrative borders and a more solid aspect [80].

Differential diagnoses of STAs encompass myonecrosis; soft tissue neoplasms, especially high-grade soft tissue sarcomas and metastases (which can demonstrate large necrotic content); hematoma; and gout tophus [81,82,83]. These conditions usually display thicker peripheral enhancement than abscesses and the central necrosis never restricts on diffusion weighted imaging, whereas thick pus inside abscess frequently can. Additionally, patient with abscesses generally present with symptoms of infection and inflammatory surrounding tissues, although those features are not specific.

Ultrasonography and CT scan can help sample the STA for microbiological analysis and target the percutaneous drainage, in addition to antibiotics adapted to the pathogen.

### 2.5. Infectious Myositis

Pyomyositis is a purulent bacterial infection of skeletal muscle that is complicated by abscess formation. Its prevalence is increasing, accounting for up to 1 case in every 3000–4000 annual hospital admissions [84,85].

Some authors distinguish the etiology of pyomyositis: a primary pyomyositis is an infection of the skeletal muscle itself caused by the hematogenous spread of microorganisms, and a secondary pyomyositis is when the infection of the muscle is by contiguity from adjacent structures, such as bone, joint, or soft tissue [84].

Most commonly, infectious pyomyositis is caused by bacteria, predominantly Staphylococcus aureus, whereas tuberculous and nonbacterial pyomyositis caused by virus, fungi, and parasites are rare [86].

Community-acquired Methicillin resistant Staphylococcus aureus (CA-MRSA) is an emergent infectious agent found not only in children, but also in the adult population [87]. 

The virulence of this pathogen may in part be related to its ability to produce toxins, such as Panton–Valentine leukocidin (PVL) which has a unique ability to kill leukocytes, resulting in bacterial evasion of the bactericidal function of leukocytes [88].

Symptoms such as limping, hip pain, and fever can be subtle in the initial stages, and this can lead to a delayed diagnosis. Pelvic muscles, thighs, and calves are the most common locations [89,90].

Pelvic location is typical in children > 10 years; the initial focus of bone infection is usually subtle, localized in a “metaphyseal-equivalent” region and has a high association with soft tissue abnormalities [91].

Ultrasound can be the first diagnostic step, especially in children, to evaluate other causes of limping, such as septic arthritis, but sensitivity is low in detecting a deep abscess and in the early stages of the infection.

Contrast-enhanced CT can show enlarged muscles, with heterogeneous attenuation and a central fluid collection with rim contrast enhancement, but it is not accurate in the early stages of pyomyositis and in the detection of infectious bone involvement [90].

**Figure 7:**
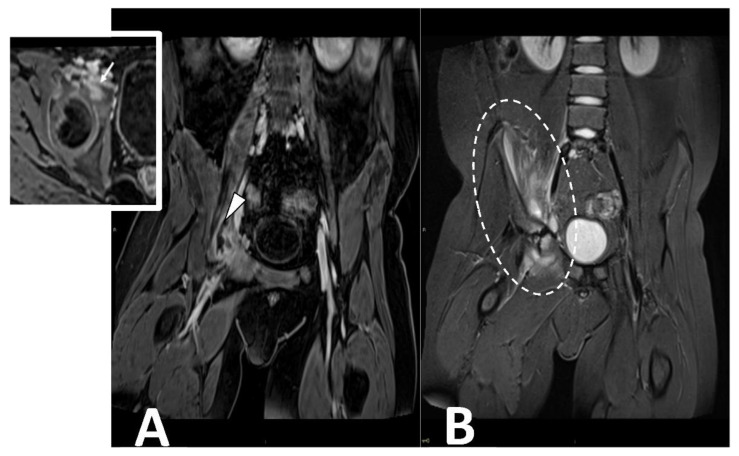
CE MRI in a 10-year-old boy presenting with limping and fever. Coronal ad axial T1 vibe FS post-contrast images (Panel **A**) demonstrate a bone infectious focus in the acetabulm (arrow) and a large abscess in the iliac and pettineus muscle with a peripheral rim enhancement (arrowhead). Coronal stir (Panel **B**) shows bone marrow edema in the acetabulum with enlargement and inflammation of surrounding muscular structures (oval dotted line).

Contrast-enhanced MRI is the gold standard in evaluating pyomyositis (Figure 7).

In the initial phlegmonous stage, MRI demonstrates an increased volume of the muscle and a non-specific increased signal on T2W images with a loss of the normal muscular architecture. It is also more accurate in the detection of a bone infectious focus [90]. The progression of the infection leads to the formation of an abscess that shows iso- and ipo- intense signals on T1-w images, a hyperintense signal on T2-w images, and a peripheral rim enhancement [92]. 

Soft-tissue sarcomas, or even bone sarcomas, may be challenging to distinguish from pyomyositis. These neoplasms usually display more defined borders, while pyomyositis often display ill-defined margins difficultly separating from surrounding edema. DWI may be helpful in discriminating between a necrotic sarcoma and an intramuscular abscess if the clinical setting is not clear. The presence of thick pus in the center of an abscess will result in a central area with restricted diffusion, while a necrotic tumor will show restricted diffusion in the peripheral wall due to high cellularity [93].

Viral myositis can be distinguished from bacterial ones on MRI. Indeed, the rarer viral forms usually present with a streaky or patchy infiltration of the muscle and an abnormally high signal on T2-weighted sequences [94]. Other possible differential diagnosis is with primary soft-tissue intramuscular lymphomas, which are characterized by a more homogenous signal than pyomyositis, as well as a very homogeneous enhancement after intravenous gadolinium injection [95]. Since pyomyositis may display, especially in the invasive stage, a wide range of clinical and radiological features, several other conditions may mimic this disease, including deep vein thrombosis, cellulitis, muscle injury/contusion/hematoma, septic arthritis, osteomyelitis, polymyositis, spontaneous gangrenous myositis trichinosis, cysticercus cellulose, and leptospirosis. 

### 2.6. Infectious Tenosynovitis and Bursitis

The term tenosynovitis refers to the inflammation of the tendon sheath, and, if secondary to an infection, it can be an orthopedic emergency. The tendon sheath is a fluid containing a closed compartment that may be predisposed to infectious diseases. Tenosynovitis can be caused by inflammation, trauma, and less frequent infections [2]. Infectious tenosynovitis can result in permanent disability with contracture of the involved muscular structures [96]. Because of this, the correct diagnosis of this entity and the differentiation from non-infectious causes are crucial, but are nonetheless challenging. The diagnosis of infectious tenosynovitis is fundamental to starting proper treatments and avoiding permanent disability.

One of the most common types of infectious tenosynovitis is the involvement of the flexor digit sheaths, usually secondary to a skin trauma that introduces a pathogen microorganism. A common complication of infectious tenosynovitis is the development of an abscess, mainly pyogenic tenosynovitis [2,96]. The microorganisms most commonly involved in infectious tenosynovitis are Staphylococcus aureus above all, followed by other bacteria, such as Pasteurella multocida (usually in cat bites), Neisseria gonorrhoeae (sexually transmitted), and Elkenella corodens (usually in human bites) [97,98].

**Figure 8:**
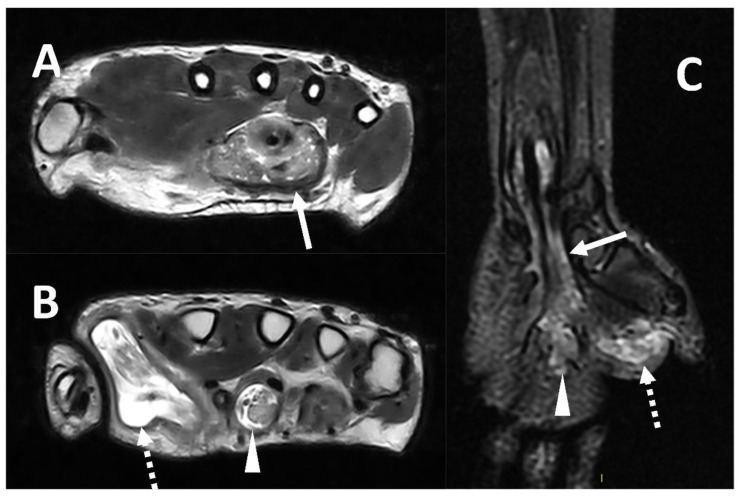
MRI (Panel **A** and **B**, T2w axial; Panel **C**, T2w coronal sequence) of a 39-year-old male complaining of progressively increasing swelling of the right wrist and hand shows a marked tenosynovitis involving the common flexor digitorum sheaths (arrows), as well as the flexor pollicis longus (dotted arrows) and the flexor of the third digit (arrowhead). Both synovial thickening and fluid collection can be detected. The final diagnosis is tenosynovitis related to mycobacterium tuberculosis.

Mycobacteria tuberculosis can also lead to infectious tenosynovitis (Figure 8), usually with an insidious clinical presentation characterized by gradual swelling and inconsistent supportive exam findings [4,97]. 

Other atypical mycobacteria tuberculosis infections can also occur, especially Mycobacteria marinum from exposure to open wounds in the sea [4,97].

Conventional radiography can be used to exclude the presence of retained foreign bodies, or, in the cases of delayed diagnosis, they can show osseous involvement (erosions and/or periosteal reactions) [4]. US is a useful tool, and it can show abnormal synovial hyperplasia, as well as increased fluid inside the tendon sheaths (usually complex with debris). Hypervascularity as examined using a Power-Color-Doppler mode evaluation may be present or not [99]. US-guided procedures (biopsy and drainage) are the preferred modalities to confirm the diagnosis and to detect the pathogen involved.

CT, especially with contrast media injection, can show the thickening of the tendon, fluid collections, or abscess. MRI is, above all, the most sensitive and specific imaging tool for infectious tenosynovitis diagnosis and evaluation, with higher contrast resolution compared to contrast-enhanced CT. MRI can directly detect the increased fluid collection within the tendon sheaths, and can offer a good characterization of synovial hyperplasia. Contrast-enhanced MRI (gadolinium) can show paratendinous contrast enhancement due to diffuse inflammation [4].

This disease may also be present on MRI with the so-called ‘rice bodies’ appearance. This non-specific MRI appearance can be encountered, especially in tuberculous tenosynovitis as well as in rheumatoid tenosynovitis. 

Differential diagnoses include inflammatory tenosynovitis, idiopathic ‘rice bodies’ tenosynovitis, pigmented villonodular synovitis, and synovial chondromatosis. The presence of gas, ulcer, skin breach, overlying cellulitis/soft tissue swelling, signs of associated septic arthritis, or foreign body should give rise to the suspicion of infectious tenosynovitis. In many cases, the diagnosis is not feasible with imaging alone, and US-guided biopsy/fluid aspiration with pathological and microbiological analysis is the only tool being able confirm the condition.

Septic bursitis is a clinical entity with quite similar clinical and imaging characteristics to infectious tenosynovitis, and it usually affects subcutaneous bursae, such as the prepatellar and olecranon ones [100]. Similar to infectious tenosynovitis, the pathogen most commonly involved in septic bursitis is Staphylococcus aureus. Due to the usually superficial location of the disease, US can be used in most of the cases as the sole imaging modality for diagnosis, guidance for drainage, and biopsy, as well as follow-up controls.

## 3. Conclusions

Imaging is a key element in the diagnosis, follow-up, and management of musculoskeletal soft-tissue infections. MRI is considered the most comprehensive and sensitive imaging tool available for many musculoskeletal infections, aiding in the assessment of all soft tissues and bones with great detail and optimal contrast [101]. US is a fundamental tool that is especially useful for the evaluation of superficially located diseases, and for US-guided procedures, such as biopsy and needle-aspiration/drainage. Conventional radiographs, even with limited/poor evaluation of soft tissues, can be very helpful in some specific conditions, above all for the first detection of foreign bodies.

Recent research demonstrates that, in some specific cases of musculoskeletal infections, imaging tools can also be used for the detection of the supposed specific microorganisms involved in the infections, with the possibility of more personalized treatments [101,102,103]. This can be helpful, especially if the pathogens are not identified in microbiological specimen analyses (usually in chronic cases). Novel quantitative applications of medical imaging (e.g., diffusion weighted MR imaging, dynamic contrast enhanced MRI, and radiomics analyses) are promising tools in this field [104,105]. Indeed, these tools could increase the sensitivity in the specific diagnoses of musculoskeletal infections, and could provide more sensitive treatment response evaluations.

## Figures and Tables

**Table 1:** Summary of imaging findings of necrotizing fasciitis.

Modality	Key Findings
Plain radiographs	• Soft tissue edema • Gas tracking along fascial planes
US	• Increased echogenicity and thickening of subcutaneous soft tissue • Fluid tracking along the fascia • Gas seen as echogenic foci with dirty posterior acoustic shadowing
CT	• Increased signal and stranding of fat (similar to cellulitis) • Deep fascial thickening and fluid along deep and intermuscular fascia • Fluid extending along intermuscular fascial planes • Lack of enhancement of fascia after intravenous contrast • Gas in soft tissue along fascial planes
MRI	• Thickening of deep fascia ≥ 3 mm • Fluid extending deep along intermuscular fascial planes • Involvement of more than 3 compartments • Variable enhancement with areas of fascial enhancement (inflammation) and lack thereof (necrosis) • Gas seen as foci of signal void on all sequences • May be band like edema/enhancement in periphery of muscles

**Table 2:** Ultrasound-guided foreign body removal step-by-step instructions.

1	Localize foreign body with high frequency linear-array transducer.
2	Perform color/power Doppler evaluation to visualize adjacent vessels and identify other critical structures at risk for injury (e.g., nerves).
3	Identify the ideal path to the foreign body and mark the skin surface accordingly.
4	Sterilely prep and drape the skin surface and place sterile cover on ultrasound probe.
5	Under ultrasound-guidance, administer 1% lidocaine to skin surface with a 25G needle along the path to the foreign body (using a 22G spinal needle if necessary).
6	Make a dermatotomy with an 11-blade scalpel.
7	Insert sterile forceps and use ultrasound to guide to the foreign body.
8	Grasp the foreign body with the forceps under ultrasound guidance.
9	Remove the foreign body through the dermatotomy site.
10	Use ultrasound to evaluate the soft tissues for additional foreign bodies or debris.
11	Clean the skin surface; a small dermatotomy may be allowed to heal by secondary intention; larger incisions can be closed with topical adhesives or adhesive strips.

**Table 3:** Summary of foreign body imaging appearances.

Foreign Body	Notes	CR	CT	US	MRI
Glass	9–24% of FBs 15% of glass injuries have retained FB	Radiopaque Nearly 100% detectable when >2 mm in size	500–1900 HU	Hyperechoic with posterior reverberation	Low T1 and T2; polygonal with angled margins
Metal	Common with gun-related injuries	Radiopaque	>3000 HU	Hyperechoic with posterior reverberation	Magnetic susceptibility artifact
Wood	36% of FBs in hand injuries Only 25% of patients note penetrating injury	Radiolucent	50–80 HU	Hyperechoic with posterior acoustic shadow; possible reverberation related to gas content	Low T1 and T2; surrounding inflammatory change and enhancement
Plastic	Uncommon as plastic rarely shatters	Radiolucent	10–20 HU	Hyperechoic with posterior reverberation	Low T1 and T2; only detects 50%; no FB reaction
Stones	e.g., asphalt or gravel after fall	Radiopaque	>1500 HU	Hyperechoic with posterior acoustic shadow	Low T1 and T2
Calcified Biologics	e.g., sea urchin spine, bones, and teeth	Radiopaque	300–1900 but decreases as resorbs over time	Thin, linear, and hyperechoic	Low T1 and T2
Non-calcified biologics	e.g., larvae; up to 9% of dermatosis in tropics	Radiolucent	Wide (lung) windows to ID respiratory tract	Echogenic; may have movement in case of larvae	Soft-tissue inflammatory mass

FB = foreign body; CR = conventional radiography; CT = computed tomography; US = ultrasound; MRI = magnetic resonance imaging.

## Data Availability

Not applicable.
